# Early automated detection system for skin cancer diagnosis using artificial intelligent techniques

**DOI:** 10.1038/s41598-024-59783-0

**Published:** 2024-04-28

**Authors:** Nourelhoda M. Mahmoud, Ahmed M. Soliman

**Affiliations:** 1 Biomedical Engineering Department, Faculty of Engineering, Minia University, Minya, Egypt; 2 Biomedical Engineering Department, Faculty of Engineering, Helwan University, Cairo, Egypt

**Keywords:** Skin cancer, Artificial intelligent, Artificial neural networks, Support vector machine, Adaptive snake, Region growing, Biomedical engineering, Skin cancer, Diagnosis

## Abstract

Recently, skin cancer is one of the spread and dangerous cancers around the world. Early detection of skin cancer can reduce mortality. Traditional methods for skin cancer detection are painful, time-consuming, expensive, and may cause the disease to spread out. Dermoscopy is used for noninvasive diagnosis of skin cancer. Artificial Intelligence (AI) plays a vital role in diseases’ diagnosis especially in biomedical engineering field. The automated detection systems based on AI reduce the complications in the traditional methods and can improve skin cancer’s diagnosis rate. In this paper, automated early detection system for skin cancer dermoscopic images using artificial intelligent is presented. Adaptive snake (AS) and region growing (RG) algorithms are used for automated segmentation and compared with each other. The results show that AS is accurate and efficient (accuracy = 96%) more than RG algorithm (accuracy = 90%). Artificial Neural networks (ANN) and support vector machine (SVM) algorithms are used for automated classification compared with each other. The proposed system with ANN algorithm shows high accuracy (94%), precision (96%), specificity (95.83%), sensitivity (recall) (92.30%), and F1-score (0.94). The proposed system is easy to use, time consuming, enables patients to make early detection for skin cancer and has high efficiency.

## Introduction

In the last decades, skin cancer is considered one of the most common and spread cancers around the world. It is extremely important to detect skin cancer in the early stage to reduce mortality^1,2^. The skin protects the body from infection, viruses such as coronavirus^3^, heat and dangerous UV radiation^4–6^. It also can store water and fat, maintain body temperature, and form vitamin D^7,8^. The estimation of the World Health Organization is recorded as approximately 132,000 skin melanoma cases per year^9^. Middle East has the highest skin cancer rates, Egypt has 1.52 rate, and its world rank is 117^10^.

Skin consists of two basic layers; the top layer is called epidermis which is made of flat cells called squamous cells. Basal cells are under the squamous cell, they are round cells. Among the basal cells, there are melanocytes cells which are responsible for pigment for skin color. Under the epidermis, there is dermis which contains blood cells and glands such as sweat gland^8^. Skin cancer is the abnormal growth of skin cells. This growth can be benign, or it can be malignant such as melanoma. Types of skin cancer are classified according to the type of cells that are subjected to cancer itself. Melanoma skin cancer, Basal skin cancer, and Squamous skin cancer are the most common types of skin cancer^11,12^. Melanoma skin cancer is the most fatal and dangerous type of skin cancer. It originated from melanocytes on the skin surface^13,14^. Basal skin cancer is originated from basal cells. Squamous skin cancer originated from Squamous cells^15^.

There are many methods to detect skin cancer. Traditional methods for skin cancer detection such as BIOPSY and naked eye^16,17^ (visual inspection by dermatologists or general practitioners), have several challenges and limitations. BIOPSY is an invasive detection method, it is painful way and time-consuming method, it also may cause the disease to spread out^18–20^. In cases where a lesion appears suspicious, BIOPSY is required for definitive diagnosis, it may cause scarring, infection, and discomfort for the patient, also it can be expensive. The naked eye is another method where doctors use their eyes and experience to determine if there is a cancer or not. Visual inspection relies heavily on the expertise and subjective judgment of the healthcare provider. The same lesion may be interpreted differently by different practitioners, leading to variability in diagnosis. Errors can occur even when skilled professionals diagnose skin lesions. It can result in both overdiagnosis (identifying benign lesions as malignant) and underdiagnosis (missing malignant lesions), therefore, visual inspection method is a non-dependable way^11,21–23^. Inaccuracy in differentiating lesions is considered one of traditional methods limitations. Distinguishing between benign and malignant lesions based solely on visual inspection can be challenging, especially for lesions that exhibit atypical features or are in early stages of development. In addition, another limitation is accessibility to dermatologists or specialized healthcare providers in skin cancer diagnosis in many regions, especially in rural or underserved areas. This limitation can lead to delays in diagnosis and treatment. To overcome these limitations, there is ongoing research and development of technologies such as dermoscopy, teledermatology and computer aided diagnosis systems, which aim to improve the accuracy, efficiency, and accessibility of skin cancer detection. Dermoscopy technique is used for diagnosing skin cancer, it is a noninvasive skin imaging technique. It acquires a magnified image of a region of skin. It has higher accuracy than evaluation by naked eyes^22,24^.

Digital skin cancer microscopic images can be improved by Machine Learning (ML)^25–27^ and Deep Learning (DL)^28,29^ techniques. Artificial intelligence and adaptation of the technology to the human service are used for different diseases detection^30–34^. The computer-based detection systems can improve the diagnosis rate of skin cancer in comparison with the traditional methods. The computer aided diagnoses system is identifying the skin images and detects the skin cancer. Image segmentation, features extraction/selection and lesion classification are used for analyzing the automated dermoscopic images^30,35,36^.

The purpose of the proposed paper is implementing automated skin cancer detection system using dermoscopic images to identify benign and malignant skin lesions using AI. In this paper, automated segmentation algorithms based on AS and RG algorithms are proposed and compared with each other. Automated classification algorithms based on ANN and SVM algorithms are proposed and compared with each other. ANN algorithm is employed to identify the most discriminative features for benign and malignant skin lesion classification to improve classification accuracy. System efficiency is evaluated using the following metrics: accuracy, specificity, precision, recall and F1-score are presented. The proposed system is easy to use, time consuming and enables patients to make early detection for skin cancer. GUI for this system is implemented.

## Related work

There are many researches in automatic detection for skin cancer with different methods and techniques. In this section, the detailed analysis of related work, including preprocessing, segmentation, and classification is illustrated.

Kang Hao Cheong et al.^23^ proposed an automated skin melanoma detection system with melanoma-index based on entropy features. The system used image pre-processing, image enhancement, entropy and energy feature mining. 600 benign and 600 digital dermoscopy malignant images from benchmark databases were examined. The classification performance assessment with the combination of Support Vector Machine (SVM) and Radial Basis Function (RBF) offered a classification accuracy about 97.50%.

Lyer et al.^37^ developed hybrid quantum mechanical system to classify cancerous and non-cancerous pigmented skin-lesions. The hybrid approach consisted of quantum classification and classical optimization using gradient descent methods. HAM10000 dataset was used, and the system achieved accuracies of 52% for training and 60% for validation.

Arora et al.^38^ developed computer-aided detection and diagnosis systems for classifying a lesion into cancer or non-cancer. They proposed feature extractor and quadratic support vector machine for skin lesions classification. The PH^2^ dataset was used, and the model achieved accuracy of 85.7%, sensitivity of 100%, specificity of 60% and training time of 0.8507 S.

Senan et al.^39^ applied the ABCD (Asymmetry, Border, Color and Diameter) rules for automatic skin cancer detection. They used PH^2^ standard dataset. Gaussian filter was applied to enhance the images. The contour method was applied for extracting the Region of Interest (RoI) from dermoscopy images. Morphology method was applied for increasing the quality of skin lesions. The ABCD rules were implemented for features extraction. Accuracy, specificity and sensitivity of the system were calculated. Accuracy was 84%, specificity was 89.50% and sensitivity was 60.50%.

Rajib Chakravorty et al.^40^ introduced a system for improving asymmetry classification in PH^2^ database using dermatologist-like feature extraction from skin lesion. Early diagnosis of melanoma was performed using asymmetry according to medical algorithms such as ABCD and CASH. They presented the performance of several classifiers using these features on PH^2^ dataset. The obtained result shows better asymmetry classification than available literature. The results for full asymmetry using SVM were accuracy (81%), precision (63%), recall (62%), specificity (87%) and F1-score (0.87).

Alan Lima et al.^41^ evaluated the melanoma diagnosis using deep features. Different classifiers were tested with the characteristic vectors extracted by the networks on the dataset PH^2^ Database. Combination of the VGG19 network and Logistic Regression (LR) was applied. The results showed accuracy (92.5%), precision (85.71%), recall (75%), specificity (96.88%) and F1-score (0.80). When applying VGG19 network and SVM linear, the results were accuracy (90.5%), precision (81.82%), recall (67.50%), specificity (96.25%) and F1-score (0.7397).

Vasconcelos et al.^42^ performed analysis of skin lesion images using principal axes-based asymmetry assessment for both dermoscopic and mobile acquired images. Two databases dermoscopic images set were used. The first is the CD-ROM Interactive Atlas of Dermoscopy and the second is the PH^2^ database. For dermoscopic images, the developed methodology using SVM achieved high accuracy (83.1%), sensitivity (75.8%), specificity (88.1%), roc (82%). While, for mobile acquired images the accuracy reached 73.1%.

Barata et al.^43^ developed two systems for the detection of melanomas in dermoscopy images using texture and color features. The best system was determined. Data set from Hospital Pedro Hispano Matosinhos was used. Both methods achieved very good results. Sensitivity was 96% and specificity was 80% for global methods using AdaBoost algorithm. Sensitivity was 100% and specificity was 75% for local methods using histogram and KNN algorithms.

Zhang et al.^44^ developed an intelligent decision support system for skin cancer detection from dermoscopic images. The system was developed to identify malignant and benigin using genetic algorithm and SVM. Dermofit dataset was used. The results showed accuracy (88%), recall (83%) and specificity (89%).

Vasconcelos et al.^45^ applied Deep Convolutional Neural Networks using small amount of data of ISBI dataset for melanoma detection. The results showed that the proposed approach improved the final classifier invariance for common melanoma variations, common skin patterns and markers, and dermatoscope capturing conditions. The results were accuracy (83.6%), precision (69.9%), recall (76%) and specificity (86.5%).

Lopez et al.^46^ introduced deep learning techniques for skin lesion classification from ISIC dermoscopic images. The proposed system determined whether the lesion was a melanoma or a benign lesion. The system was built using the VGGNet convolutional neural network architecture and used the transfer learning paradigm. The results were accuracy (81.33%), precision (79.74%) and recall (78.66%).

## Materials and methods

This paper proposed an intelligent system for skin cancer detection. The implemented system is developed to detect benign and malignant skin lesions. Multiple steps, including pre-processing, different methods for segmentation, features extraction/features selection, and different methods of classification are used for analyzing the automated dermoscopic images.

**Figure 1:**
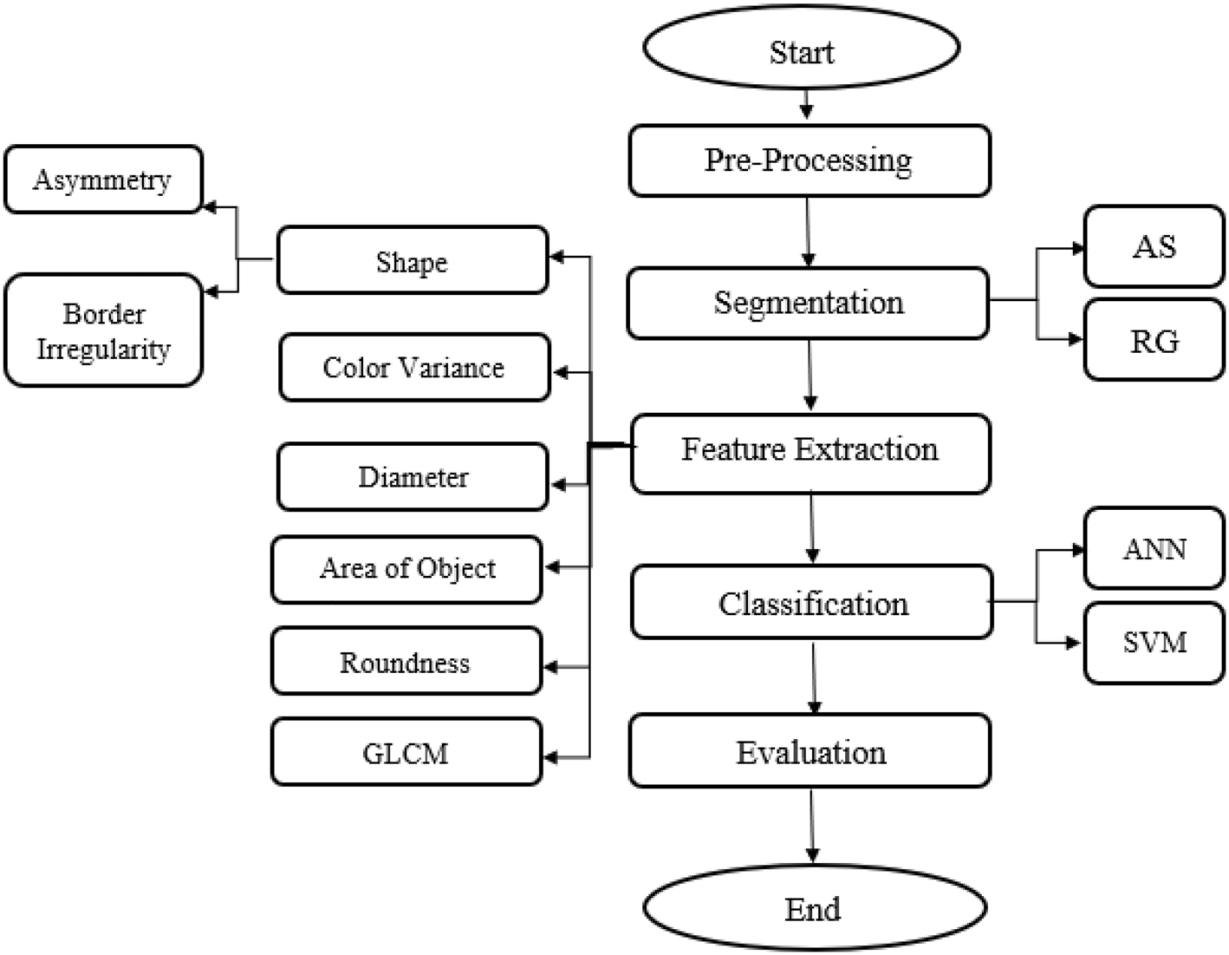
Flowchart for the implemented proposed system.

The dermoscopic images dataset used in this paper is from PH^2^ Dataset^47^. This dataset is publicly available^47^. PH^2^ Dataset is a widely used dataset in the field of dermatology and skin cancer detection. PH^2^ dataset contains dermoscopic images that were obtained at the Dermatology Service of Hospital Pedro Hispano (Matosinhos, Portugal) under the same conditions through Tuebinger Mole Analyzer system using 20× magnification. The dermoscopic images are 8-bit RGB color images with a resolution of 768 × 560 pixels^47^. PH^2^ Dataset is a widely used dataset in the field of dermatology and skin cancer detection. PH^2^ dataset contains three types of skin diseases, they are Atypical Nevi, Melanoma and Common Nevus. This dataset includes 200 dermoscopic images (80 common nevus, 80 atypical nevi, and 40 melanomas). The dermoscopic images were selected randomly for training and test, as 80% for training and 20% for test. GUI for this study was implemented. The system configuration used for the proposed models was Intel Core i5 with processor 1.80 GHz and 8 GB of RAM. The system was implemented using MATLAB program. Figure 1 shows the flowchart of the implemented proposed system.

1. Pre-processing

Each image has several noises, therefore, the noise that appeared in the images should be eradicated for improving identification process. Pre-processing includes reading the image and applying sequence of hair removal filters using DullRazor algorithm^48^; negative the image; enhancing the contrast of image; gray scale transformation; converting it to grayscale; noise filtering by median filter; and finding the negative of image by finding maximum of this image. The proposed system is characterized by using series of filters to enhance the contrast of the image. These filters are median, Gaussian and lee filters. Negative of enhanced image is applied. Smooth images are obtained after pre-processing step.

2. Segmentation

Segmentation is a technique to separate the objects from their background. It can adapt to the complex morphology of biological structures. In the step of segmentation, Adaptive snake and Region growing algorithms are used and modified.

In the segmentation process for skin cancer detection, AS and RG algorithms offer valuable advantages including efficiency, flexibility, incorporation of prior knowledge, adaptation to image variability, and exploitation of local homogeneity. The choice between these two algorithms is dependent on factors such as the complexity of the lesion, the quality of the input images, and computational resources available.

*Segmentation using adaptive snake*^49^ includes finding the size of interested image; creating a mask of zeros by the same size of the interested image; assigning one value for a specific region in the created mask to snake around lesion; resizing all images to be the same; and applying the mask on the interested image. The result will be the interesting lesion.

AS algorithm is deformable model that can adapt to the contours of skin lesions. This flexibility enables AS to accurately identify irregularly shaped lesions, which is common in skin cancer cases. In addition, AS often incorporate prior knowledge about the shape and appearance of skin lesions, including color, texture, and gradient information. It can help improve the accuracy of segmentation by guiding the snake towards relevant features. Their shape and position based on the characteristics of the image can be adjusted, such as intensity gradients and edges. This flexibility makes AS robust to variations in image quality, lighting conditions, and skin types.

*Segmentation using region growing*^50^ includes finding the size of interested image; specifying the lesion coordinates; determining the point form where the machine begin to grow by knowing X & Y Coordinates; applying opening and closing morphological methods.

RG starts from one or more seed points within the lesion and iteratively grows the region by adding adjacent pixels that satisfy certain criteria, such as similarity in intensity or color. This seed-based approach allows for the segmentation of lesions without requiring explicit boundary information. RG exploits the concept of local homogeneity within skin lesions, meaning that pixels within the lesion exhibit similar characteristics in terms of color, texture, or intensity. Therefore, even in the presence of noise or variations in illumination, RG algorithms can accurately segment lesions. In addition, it is computationally efficient and can produce segmentation results quickly, making them suitable for real-time or large-scale applications. Compared to other traditional image segmentation techniques, such as thresholding or clustering, RG offers unique characteristics and advantages including boundary sensitivity and it considers local pixel relationships and can capture fine-grained details and complex boundaries.

The evaluation of AS and RG algorithms is achieved using accuracy as evaluation metric. Accuracy measures the overall correctness of the segmentation results, considering both true positive and true negative pixels.

3. Features extraction

The next important step is deriving the features from the affected region. The extracted feature is reflection of the affected area information^51^.

Gray Level Co-occurrence matrix (GLCM) is used for features extraction. GLCM is a statistical method to examine and characterize the texture of the image. It is a second-order statistical texture analysis method. It is also known as the gray-level spatial dependence matrix^52–55^.

GLCM Parameters include Autocorrelation, Contrast, Correlation, Cluster-Prominence, Cluster Shade, Dissimilarity, Energy, Entropy, Homogeneity, Maximum probability, Sum of squares, Sum average, Sum variance, Sum entropy, Difference variance, Difference entropy, Information measure of correlation1, Information measure of correlation, Inverse difference normalized (INN), Inverse difference moment normalized^52–55^.

ABCDE rules of dermatology are used for features extraction. ABCDE rules are the commonly approved approaches for skin lesions detection. The selected features are shape including asymmetry and border irregularity, color variance, diameter, area of object, and roundness^39,56,57^.

ABCDE rules for features extraction are as the following:

1. Shape
  - Asymmetry: The lesion asymmetry was evaluated by calculating the area with inner and outer of the lesion, as follows^58,59^:
    1 $$ASI= \frac{\Delta AK}{AL}\times 100$$
    where, *ASI* is asymmetry index. *ΔAK* is the area between the two halves of the lesion and *AL* is lesion area.
  - Border irregularity: The edge of a malignant lesion usually exhibits four factors of interest, density, fractal dimension, radial variability and the extent to which its contour exhibits small irregularities. Border Irregularity is evaluated as follows^60^:
    2 $$I= \frac{ab}{2\pi (a2+b2)}\frac{p2}{\Delta A}$$
    where, *I* denotes irregularity with *a* and *b* representing the lengths of major and minor axes of the lesions. *P* is the perimeter of the lesion and *ΔA* is the area of corresponding.
2. Color Variance
  This feature helps in finding the variations in colors among several types of images by converting images from RGB to HSV^56^.
3. Area of Object
  Lesion images can be classified by finding area of segmented lesion or area of interest. This can be done by converting image to black and white and segment lesion and find diameter from 'regionprops'^60^.
4. Diameter
  Lesion images can be classified by finding the diameter of segmented lesion or area of interest. This can be done by converting image to Black & White and segment lesion and find diameter from 'regionprops'. Diameter is evaluated as follows^46,51^:
  3 $$Diameter=\sqrt{4*Area \,\, of \,\,object/\pi }$$
5. Roundness
  Roundness of lesion can be calculated after finding area of object to distinguish common lesions from diseased one.
  Roundness is evaluated as follows^60^:
  4 $$Roundness=\sqrt{4*\pi *Area \,\,of \,\,object/Perimeter^{2}}$$
6. Classification

ANN and SVM algorithms are used for classification of skin cancer. Classification includes specifying all image's features; entering input data and target data; applying validation and performance on it; and using train data and test data.

ANN is also known as Neural Networks. ANN are trained using a supervised learning approach. There are three types of computational nodes. There is input layer, nodes in it have a connection with the hidden layer. A typical ANN consists of multiple hidden layers. The number of input units connected to the hidden layer depends on the dataset. Output nodes produce the final output of the neural network after receiving the processed data from the hidden layers. The number of output nodes depends on the nature of the task that the network is designed for. ANN is particularly useful for skin cancer detection because ANN is excellent in learning complex representations from raw data. It can learn complex patterns and relationships from the input data, which is crucial for accurately classifying skin lesions.

SVM is used for classification, it is supervised learning model with associated learning algorithms that analyze data and recognize patterns, used for classification and regression analysis. SVM takes a set of input data and predicts, for each given input, which of two possible classes forms the output. SVM aims to find the hyperplane that best separates different classes in the input space. It works by mapping input data into a higher-dimensional feature space and finding the optimal separating hyperplane with the maximum margin between classes. SVM is effective in skin cancer detection because it can handle high-dimensional data and is robust to overfitting. SVM can also efficiently handle nonlinear relationships in the data by using kernel functions, allowing it to classify skin lesions accurately. SVM is widely used to classify digital dermoscope images^61^.

Both ANN and SVM offer advantages for skin cancer detection. By learning from a variety of training data, ANN can adjust to variations in imaging conditions, lesion characteristics, and patient demographics. In real clinical settings, this adaptability can enhance generalization performance. Compared to other ML models, SVM is less prone to overfitting, especially when using appropriate regularization techniques and kernel functions. This robustness is helpful in the detection of skin cancer, where the ability to generalize to new data is essential. SVM can perform well even with relatively small training datasets such as PH^2^ dataset, making it suitable for skin cancer detection where collecting large amounts of labeled data may be challenging.

In the classification process of skin cancer detection, both ANN and SVM algorithms are typically trained using labeled datasets containing various features extracted from images of skin lesions, such as color, texture, and shape information. Once trained, these algorithms can classify new skin lesion images into different categories, such as benign or malignant, based on the learned patterns and relationships in the data. The choice between ANN and SVM depends on factors such as the size and complexity of the dataset, computational resources available, and the desired performance metrics. Both algorithms have demonstrated promising results in skin cancer detection and are actively used in research and clinical applications.

The accuracy of skin cancer detection depends on the efficiency of classification.

5. Evaluation

Evaluation metrics are very important in the development, validation, and deployment of skin cancer detection algorithms. They provide critical insights into algorithm performance, guiding improvements, supporting clinical decision-making, and ultimately contributing to better patient outcomes.

The efficiency of the proposed system is evaluated using the following metrics:

Accuracy: This measure records the correct and incorrect recognized samples of each class according to confusion matrix to evaluate the classification quality. A confusion matrix is a binary classification which is determined as TP: true positive, FP: false positive, FN: false negative, TN: the true negative amount(s). Accuracy is evaluated as follows^62^:

5 $$Accuracy= \frac{TP+TN}{TP+TN+FP+FN}$$

Sensitivity or Recall is called true positive rate and it measures the proportion of actual positives that are correctly identified as such (e.g., the percentage of sick people who are correctly identified as having the condition). Sensitivity is evaluated as follows^32^:

6 $$sensetivity= \frac{TP}{TP+FN}$$

Specificity is called true negative rate and it measures the proportion of actual negatives that are correctly identified as such (e.g., the percentage of healthy people who are correctly identified as not having the condition). Specificity is evaluated as follows^63^:

7 $$Specificity= \frac{TN}{FP+TN}$$

Precision is called positive predictive value. It is the fraction of relevant instances among the retrieved instances. Precision is evaluated as follows^32,63^:

8 $$Precision=\frac{ TP}{TP+FP}$$

Dice or F1 Score is a measure of a test's accuracy. It considers both the precision and the recall of the test to compute the score. F1 score will be best value at 1 when the system achieves perfect precision and recall; and F1 will be worst at 0. F1 score is evaluated as follows^23^:

9 $$F1 score=Dice=2.\frac{precision.recall}{precision+recall}$$

The Jaccard index is known as Intersection over Union (IoU) and the Jaccard similarity coefficient. It is a statistic used for comparing the similarity and diversity of sample sets. The Jaccard coefficient measures similarity between finite sample sets. It is defined as the size of the intersection divided by the size of the union of the sample sets. Jaccard is evaluated as follows^64^:

10 $$Jaccard=\frac{F1}{2-F1}$$

Matthews correlation coefficient (MCC) is calculated as follows^65^:

11 $$MCC= \frac{\left(TP.TN\right)-(FP.FN)}{\sqrt{\left(TP+FP\right).\left(TP+FN\right).\left(TN+FP\right).(TN+FN)}}$$

## Results and discussion

In this section, the results obtained are presented. The results from the proposed methodology compared with the previously published research.

1. Segmentation

**Figure 2:**
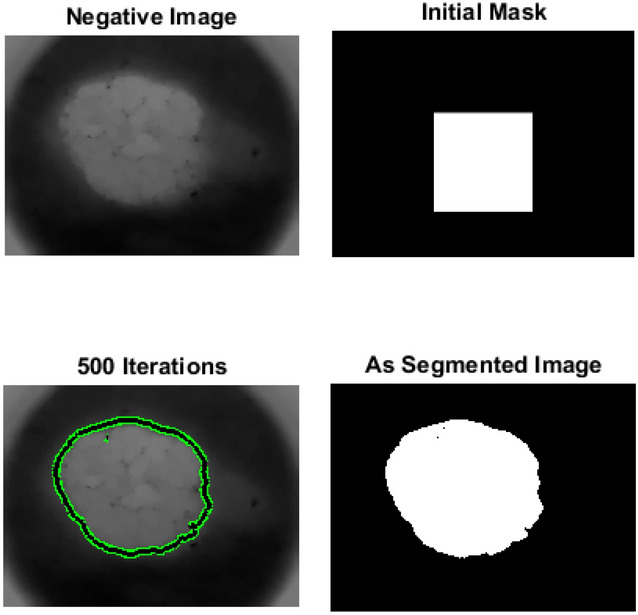
Adaptive snake segmentation.

**Figure 3:**
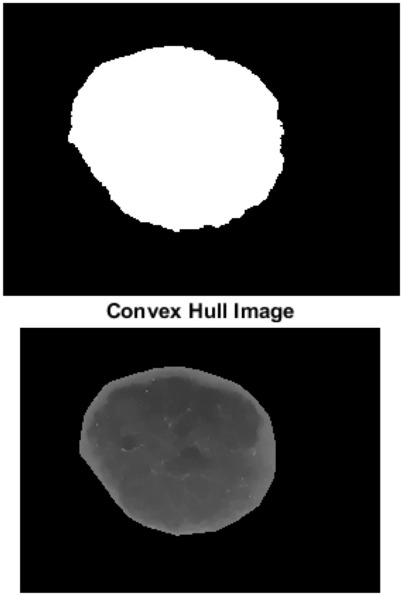
Region growing segmentation.

AS and RG algorithms are used for segmentation. Figure 2 shows segmentation using AS, while Fig. 3 shows segmentation using RG. Accuracy for AS is 96% while accuracy for RG is 90%.

Results show that both AS and RG have their strengths in the segmentation process for skin cancer detection. AS is more efficient than RG due to its flexibility, ability to incorporate prior knowledge and ability to handle complex lesions. AS provides more precise boundaries compared to RG, especially for objects with irregular boundaries or complex shapes. AS is more suitable for a wider range of images. Compared to RG, AS optimizes the contour globally based on the entire image.

It is clear that, AS is simple, more accurate and segment specified region, but it needs to specify mask size. RG can segment image if it is symmetric, but it is slower than AS and it can’t segment image if it is Asymmetric.

2. Classification using NN and SVM

The classification results are presented in terms of seven metrics: precision, accuracy, sensitivity, specificity, F1 score, jaccard and MCC.

**Figure 4:**
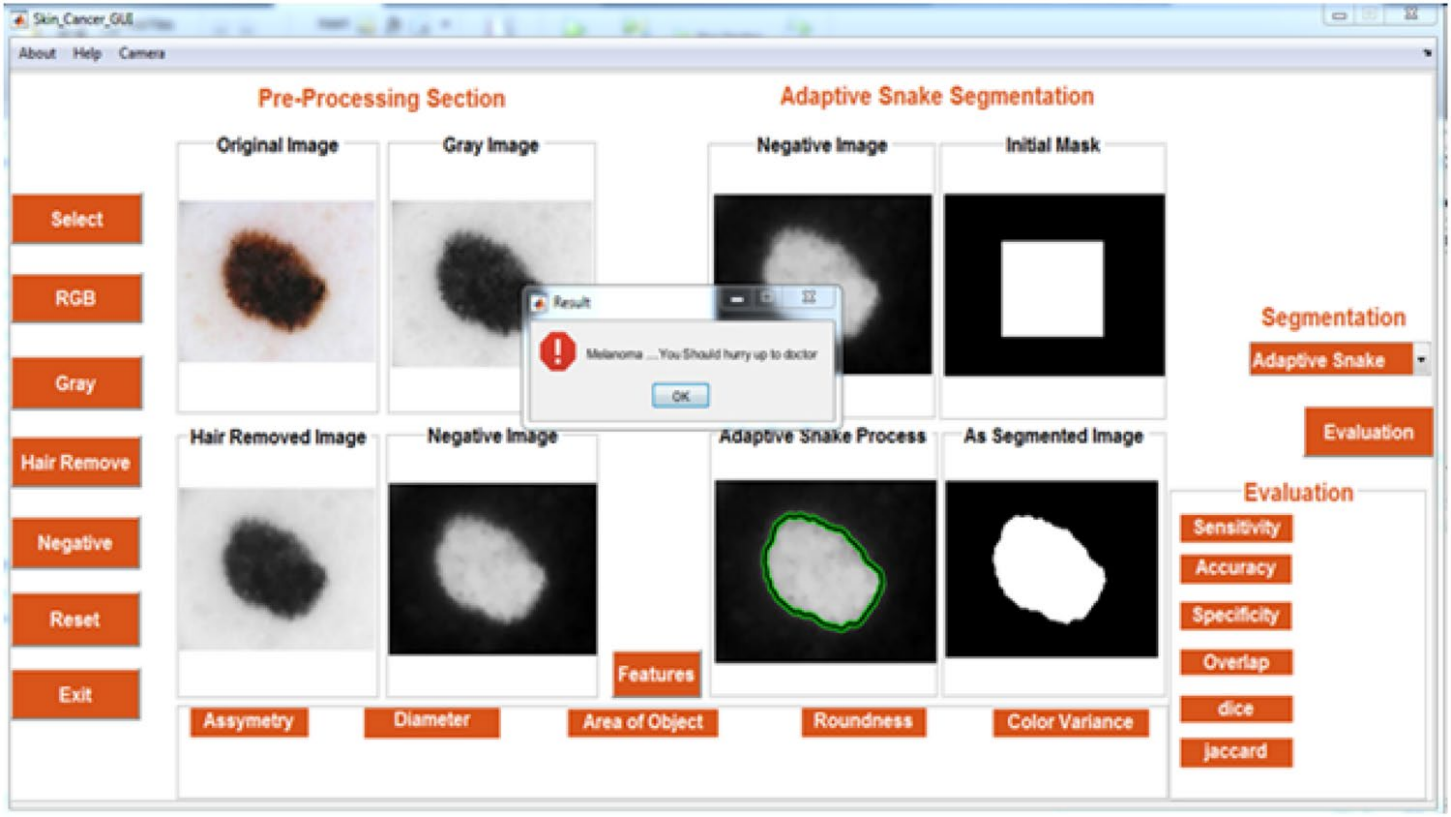
GUI for the proposed system.

Figure 4 shows GUI for the proposed system. Table 1 summarizes the performances obtained by the proposed system in comparison with the results from literature review, including the methods used, datasets, algorithms and results achieved for the diagnosis of skin cancer dermatoscopic images. It is important to notice that a comparison would imply implementation of all methods and validation with the same dataset. However, it could be noticed that the obtained results are comparable with the best results in the literature.

**Table 1:** Results of the proposed system compared with results from literature review.

Paper No	Dataset	Algorithm	Percision %	Accuracy %	Sensitivity = Recall %	Specificity %	Dice = F1 Score	Jaccard	MCC %
Proposed work	PH^2^	ANN	96	94	92.30	95.83	0.94	0.886	88.5
Proposed work	PH^2^	SVM	76	74	73.07	75	0.74	0.587	48
37	HAM10000	2-qubit hybrid quantum model	NA	52	NA	NA	NA	NA	NA
38	PH^2^	SVM	NA	85.7	100	60	NA	NA	NA
39	PH^2^	ABCD rule	NA	84	60.50	89.50	NA	NA	NA
40	PH^2^	SVM	63	81	62	87	0.87	NA	NA
41	PH^2^	VGG19 + LR	85.71	92.5	75	96.88	0.80	NA	NA
41	PH^2^	VGG19 + SVM linear	81.82	90.5	67.50	96.25	0.7397	NA	NA
42	PH^2^	SVM + OTSU	NA	83.1	75.8	88.1	NA	NA	NA
43	Hospital Pedro Hispano (PH^2^)	AdaBoost Histogram + KNN	NA NA	NA NA	96 100	80 75	NA NA	NA NA	NA NA
44	Dermofit	GA + SVM	NA	88	83	89	NA	NA	NA
45	ISIC	Inception	69.9	83.6	76	86.5	NA	NA	NA
46	ISIC	VGG16	79.74	81.33	78.66	NA	NA	NA	NA

As shown in Table 1, the proposed system with ANN algorithm has maximum efficiency [accuracy (94%), precision (96%), specificity (95.83%), sensitivity (recall) (92.30%), and F1-score (0.94)] compared to the proposed system with SVM algorithm. Also, the proposed system with ANN algorithm has maximum efficiency compared to other research using PH2, Hospital Pedro Hispano (PH2), HAM10000, Dermofit, and ISIC datasets.

The proposed methodology outperforms the previously published result. Consistent classification performance in all the metrics across various classifiers indicates the suitability of the proposed features and methodologies.

Therefore, the proposed system is easy to use, time consuming, enables patients to monitor remotely and make early detection for skin cancer and has high efficiency. It also improves skin cancer’s diagnosis rate. Automated early detection system for skin cancer dermoscopic images using artificial intelligent accelerates the time of dermatologists and improves diagnosis performance.

## Conclusion

In this work, automated early detection system for skin cancer dermoscopic images using artificial intelligent is presented. The proposed system accelerates the time of dermatologists and improves diagnosis performance. All the images in the PH^2^ database are used, divided into 80 common nevus, 80 atypical nevi, and 40 melanomas images. The system was implemented using MATLAB program.

The proposed system is developed to detect benign and malignant skin lesions using multiple steps, including pre-processing, different methods for segmentation, features extraction/features selection, and different methods of classification are used for analyzing the automated dermoscopic images.

From the study of literature, it is concluded that various methods are employed for detecting skin cancer. The results show that AS is more accurate and efficient than RG algorithm. Accuracy for AS is 96% while accuracy for RG is 90%. Artificial Neural networks (ANN) and support vector machine (SVM) algorithms are used for automated classification which are applied and compared with each other. The proposed system with ANN classifier has high performances, accuracy (94%), precision (96%), specificity (95.83%), sensitivity (recall) (92.30%), and F1-score (0.94). The proposed system improves skin cancer’s diagnosis rate.

This study utilized two ML models (ANN and SVM); they offer advantages for skin cancer detection using the PH^2^ dataset. ANN is excellent in learning complex representations from raw data and SVM provides robustness and efficiency. The implemented proposed system is efficient, accurate and easy to use by different users (doctors and patients). Early detection and diagnosis of skin cancer can lead to more successful treatment outcomes and potentially save lives, in addition, it can reduce the overall cost of treatment.

In the future work, the proposed system can be operated on a real-time diagnosis application after obtaining required approval and declarations. Additionally, more methods such as another ML models or deep learning models will be implemented aiming to enhance the performance level. Other available datasets can be used to test the suggested framework's ability to categorize skin cancer.

## Data Availability

The dermoscopic images dataset used in this paper is from PH^2^ Dataset. This dataset is publicly available from the website: https://www.fc.up.pt/addi/ph2%20database.html.
